# Effects of passive ankle exoskeletons on neuromuscular function during exaggerated standing sway

**DOI:** 10.1098/rsos.230590

**Published:** 2024-05-01

**Authors:** Dominic J. Farris, Jemima C. N. Po, Jordan Yee, James L. Williamson, Taylor J. M. Dick

**Affiliations:** ^1^ Public Health and Sports Science, Faculty of Health and Life Sciences, University of Exeter, Exeter, UK; ^2^ Human Movement and Nutrition Sciences, Faculty of Health and Behavioural Sciences, University of Queensland, , Australia; ^3^ School of Biomedical Sciences, Faculty of Medicine, University of Queensland, , Australia

**Keywords:** exoskeleton, balance, plantar flexors, ultrasound, electromyography

## Abstract

Wearable robotic exoskeletons designed to assist human movement should integrate with the neuromusculoskeletal system. This means assisting movement while not perturbing motor control. We sought to test if passive ankle exoskeletons, which have been shown to successfully assist human gait, affect neuromuscular control of an exaggerated anterior–posterior standing sway task. Participants actively swayed while wearing an ankle exoskeleton that provided 0, 42 or 85 Nm rad^−1^ of additional stiffness to the ankle joint in resistance to dorsiflexion. Sway amplitude was controlled via biofeedback to elicit similar ankle angle displacements across conditions. With greater exoskeleton stiffness, participants swayed at lower sway-cycle frequencies and slower centre of pressure speeds. Furthermore, increasing exoskeleton stiffness resulted in longer operating lengths of the medial gastrocnemius and overall reduced plantar flexor muscle activation. For the soleus, there was also a temporal shift in the cross-correlation of its electromyogram with the centre of pressure displacement, meaning that muscle activation peaked later than anterior sway displacement. Together, these data suggest that assistive ankle exoskeletons influence neuromuscular control of ankle-based sway tasks. Changes in fascicle lengths could influence afferent feedback signals and the short-range stiffness of ankle muscles, while shifts in muscle activation timing suggest changes in neural control. The observed neuromuscular adaptations to exoskeleton assistance demonstrate the potential implications for standing balance and overall movement control, prompting future investigations.

## 1. Introduction

Assistive exoskeletons are wearable robotic devices designed to assist, restore or augment the wearer’s physical capability. Research and development of exoskeletons have significantly advanced in recent years, with applications spanning gait rehabilitation [1], manual handling [2] or body-borne load carriage [3], to name a few. A particular focus has been the design of lower limb exoskeletons intended to reduce mechanical and metabolic demands on the user, either to restore function for activities of daily living or to augment walking and running performance [4]. Notably, exoskeletons providing mechanical assistance to the ankle joints, including passive spring-loaded devices [5], have successfully lowered the energy cost of walking and running (e.g. [5–8]). Furthermore, ankle exoskeletons have been shown to reduce plantar flexor muscle activations during locomotor tasks [9,10]. However, ankle exoskeletons may have an associated unintended effect of increasing plantar flexor fascicle operating length and length changes during walking [11] and hopping [10].

Modulation of ankle muscle mechanics and activations by exoskeletons may have consequences for neuromuscular function and control of other tasks. Maintaining balance and postural control during upright standing requires constant monitoring of the neuromuscular states of ankle muscles and responsive adjustments to muscle activation [12]. Schiffman *et al*. [13] showed that a passive lower limb exoskeleton reduced the magnitude of postural sway and the limits of stability in soldiers standing with a loaded backpack. However, it is unclear if this is linked to any underlying changes in muscle function. More recently, Emmens *et al*. [14] found that an ankle exoskeleton resulted in reductions in soleus (SOL) electromyographic (EMG) activity during balance perturbation tasks, with a concomitant increase in tibialis anterior (TA) activation. The reduction in plantar flexor activation is probably owing to reduced voluntary motor neuronal drive, in response to the additional plantar flexor torque provided by the exoskeleton. Such a response maintains total ankle torque (exoskeleton plus biological) at similar levels to when no exoskeleton assistance is provided, which should allow the same kinematic response to be maintained. However, reduced plantar flexor activation and force imply a lesser stretch of the Achilles tendon [15]. If ankle rotation is preserved while tendon stretch is reduced, the muscle fascicles must operate at longer lengths, and this might alter sensorimotor feedback signals from muscle spindles and Golgi tendon organs.

The increase in TA EMG observed by Emmens *et al*. [14] is harder to explain, but the authors suggested that it could have been a voluntary response to further reduce the net plantar flexor ankle moment without making further reductions in SOL activation. This suggests that there may be a limit to which exoskeleton wearers are willing to deactivate muscles and rely on exoskeleton torque. Alternatively, increased TA EMG might be an involuntary response resulting from reduced inhibitory signals accompanying the reduction in plantar flexor activity [16]. Either mechanism supports the probability that wearing a plantar flexion-assist exoskeleton during ankle-dorsiflexion will result in increased TA EMG. Furthermore, Beck *et al*. [17] demonstrated that fast-reactive ankle exoskeleton torque can disrupt SOL muscle mechanics during balance recovery from surface translations. Based on this evidence, there is potential for exoskeletons to affect neuromuscular control of sway and balance during upright standing. The length changes in ankle plantar flexor fascicles during postural sway have been described as paradoxical: shortening during dorsiflexion (forward sway) and lengthening during plantar flexion (backward sway). Preliminary work to illustrate this principle involved exaggerated active standing sway tasks that required larger, more easily observed fascicle length changes [12]. Consequently, a similar experimental paradigm involving exaggerated sway is a good starting point for studying if exoskeletons influence fascicle length change patterns during standing balance tasks.

Therefore, the aim of this study was to assess the influence of a passive exoskeleton designed to assist ankle plantar flexion on neuromuscular function during an active standing ankle-based sway task. The task involved feedback-based targeting of centre of pressure (COP) displacement to produce a consistent magnitude of exaggerated anterior–posterior sway. We hypothesized that spring-loaded plantar flexion assistance during sway tasks would: (i) slow the rate at which individuals sway (owing to device novelty); (ii) reduce plantar flexor EMG activity to maintain total net ankle torque profiles; (iii) increase the operating length and length change of plantar flexor muscle fascicles, that must account for a greater proportion of MTU length changes when Achilles tendon stretch is reduced; and (iv) increase TA EMG activity as has been observed previously for plantar flexion-assist exoskeletons during balance tasks [14].

### 2. Methods

Data were recorded from 17 healthy individuals (mean ± s.d., 11 male, 6 female; 24.9 ± 5.1 years; 1.75 ± 0.1 m; 73.7 ± 12.6 kg) with no prior exoskeleton experience. All participants were healthy and had no recent (within 12 months) lower limb pain or injury. Participants provided written informed consent, and all protocols were approved by the institutional ethics review committee at the University of Queensland (2019002595).

#### 2.1. Experimental protocol

**Figure 1.**
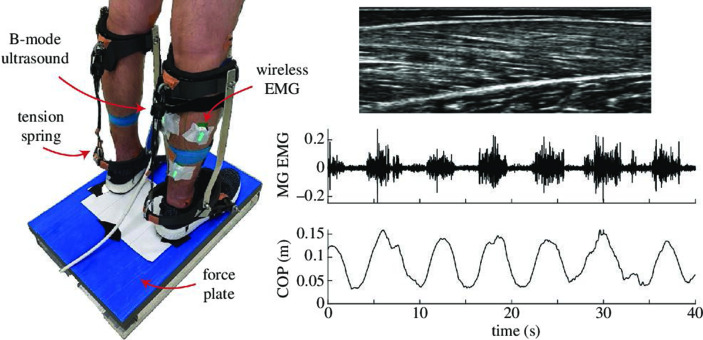
Image of the experimental set-up, sample B-mode ultrasound image of medial gastrocnemius (MG), with time-varying surface EMG and COP data.

Participants were then asked to stand in a comfortable upright position on the hard, flat surface of the force plate. For standardization and repeatability purposes, the participants’ feet were positioned with their heels 14 cm apart, and the location of both feet was traced on a piece of paper taped to the surface in order to ensure a similar standing position between trials. Participants wore custom-made bilateral passive ankle exoskeletons (figure 1) and a safety harness during the postural tasks. Participants were instructed to actively sway at their preferred frequency (0.11 ± 0.02 Hz) for 60 s under three different conditions: (i) no assistance, 0 Nm rad^−1^ (i.e. with an exoskeleton but no spring); (ii) 42 Nm rad^−1^; and (iii) 85 Nm rad^−1^. During each trial, the participants had their arms positioned across their chests, and if required between trials, their feet were repositioned to match the initial trace. To test the effect, exoskeleton stiffness (0, 42 and 85 Nm rad^−1^) conditions were randomized. Participants were given approximately 60 s of rest between trials to minimize the effects of fatigue.

Because the limits of postural sway may depend on the presence of exoskeleton assistance, participants first completed the active sway task without assistance so we could determine their average anterior and posterior limits of sway. Although sway is defined as the oscillation of the body’s centre of mass (COM) [18], body sway can be assessed by measuring changes in the location of the COP during standing balance tasks [19]. This measure does not directly represent body sway; instead, it serves as an indicator of the activity of the neuromuscular system in controlling the movement of the COP [20]. Sway was measured as the participant’s COP range of motion in the anterior–posterior direction. This self-selected amplitude was as close to the person’s limits of stability (i.e. sway position that would require a recovery step in the forwards or backwards direction to avoid a potential fall) that they could comfortably obtain and was selected as a postural task to determine the influence of ankle exoskeletons on neuromuscular function under voluntary control. These anterior and posterior sway limits were provided as visual biofeedback targets of the participants’ COP as horizontal lines on a computer monitor directly in front of them. Participants were asked to match the sway amplitude between the three conditions. We found no effect of exoskeleton stiffness on sway amplitude (*p* = 0.424) suggesting that sway amplitude remained consistent across trials for each participant.

#### 2.2. Ankle exoskeleton

An image of the exoskeleton used in this study is shown in figure 1. The exoskeleton consisted of a three-dimensional (3D) printed attachment around the upper shank which was connected to a 3D printed foot section via two aluminium bars, which had a freely rotating joint aligned with the participants’ medial and lateral malleoli. These components were 3D printed using Onyx (Markforged Inc., USA)—a micro-carbon-fibre-filled nylon material. The foot section was attached to a running shoe through the sole of the shoe inferior to the heel and towards the front of the shoe at the approximate position of the first MTP joint. Foam was attached to the shank cuff to avoid discomfort. The 3D printed attachments at the shank and the foot, the aluminium bars and the running shoes were varied in size to match the leg and foot dimensions of the participant. An extension spring could be attached superiorly to an eye bolt on the posterior aspect of the shank cuff and inferiorly on the heel of the foot segment via an aluminium link, nylon rope and a locking cam-jam (Nite Ize Inc., USA). The stiffness of the two different springs in tension was 2.5 and 5 kN m^−1^, and the moment arm about the ankle joint was 0.13 m. This provided a rotational stiffness of 42 and 85 Nm rad^−1^, respectively. The length of the rope was adjusted for each participant such that the resting length of the spring coincided with an ankle angle of neutral standing ankle angle minus 13° (i.e. 13° more plantar flexed position). This angle was chosen such that when in neutral standing, the exoskeleton would provide approximately 5% and 10% of the total ankle torque measured during standing balance tasks [21]. Owing to their initial tension, the springs generated plantar flexion torques of 9.5 and 19.3 Nm when the spring was initially stretched beyond its resting length for the 42 and 85 Nm rad^−1^ devices, respectively. From that point, further plantar flexion resulted in torque increasing according to the rotational stiffness of the respective springs.

#### 2.3. Centre of pressure

Participants stood on a single force plate to determine the location of the COP (2048 Hz; Bertec Corp., USA). Force plate data were collected via a data acquisition board (CED1401; CED Ltd, UK) and then combined with EMG and ultrasound data via a custom-written script. The COP data were filtered with a second-order low-pass Butterworth filter at 6 Hz. The position of the COP in the X direction (anteroposterior) was used to calculate sway parameters (larger values indicate forward sway and smaller values indicate backwards sway). The time-varying COP signals were divided into sway cycles based on the minimum of the COP (most posterior sway position) and averaged across cycles to determine an average COP signal for each condition to be used within the cross-correlation analysis. We also calculated sway frequency, mean COP displacement (distance during a sway cycle) and mean COP sway speeds during the full sway cycle as well as during the anterior and posterior sway directions. Sway variables were averaged across cycles over the 60 s trials for each participant. Because it is likely that the exoskeleton provides support in the frontal plane, we did not analyse the COP in the mediolateral direction.

#### 2.4. Electromyography

Surface EMG was used to record muscle activity from the medial gastrocnemius (MG), lateral gastrocnemius (LG), SOL and TA on participant’s dominant leg. We shaved, abraded and cleaned participant’s skin with alcohol to reduce the skin-electrode impedance. We placed bi-polar surface electrodes (Trigno Delsys Inc., Natick, MA, USA; 10 mm inter-electrode distance) over the MG, LG, SOL, TA muscle bellies, aligned along the direction of the muscle fascicles, as determined using B-mode ultrasound. Elastic bandages secure the electrodes to the skin to avoid movement artefacts. The EMG signals were amplified, digitized at 2048 Hz and recorded in Spike2 (v. 7; CED Ltd, Cambridge, UK). EMG signals were band-pass filtered using a second-order Butterworth filter (20–500 Hz). To determine a time-varying EMG envelope, we calculated the root mean squared (RMS) amplitude for each muscle using a 100 ms sliding window over the 60 s trials. The time-varying RMS EMG signals were divided into sway cycles based on the position of the COP and averaged across cycles to determine an average RMS EMG for each muscle at each condition to be used within the cross-correlation analysis. As a measure of global activation over the balance task, we averaged the RMS values for each muscle over the 60 s period.

#### 2.5. Ultrasound

We recorded B-mode ultrasound for the MG using a linear transducer (5–8 MHz, 60 mm field of view, LV8-5L60N-2; Telemed, Vilnius, Lithuania) coupled with a PC-based ultrasound system (ArtUS EXT-1H; Telemed). The transducer was placed in a custom-made probe holder and positioned over the muscle belly of the MG on the participants’ dominant leg using an elastic bandage. The transducer was aligned so that the MG fascicles could be visualized from the deep to the superficial aponeuroses. Ultrasound data were sampled at 125 Hz (Echo Wave II 3.7.1; Telemed) and recorded for 20 s within the middle of the 60 s active sway trial. An external trigger generated by Spike2 (v. 7; CED Ltd) was used to synchronize the ultrasound with the COP and EMG recordings. We digitized ultrasound images using semi-automated tracking software [22] to determine time-varying MG fascicle length during the active sway trials. Time-varying fascicle lengths were divided into sway cycles based on the position of the COP. Sway cycles were averaged to determine the absolute fascicle length change, maximum fascicle length and minimum fascicle length for each participant at each condition. Ultrasound data from two participants were excluded from the analysis owing to poor image quality.

#### 2.6. Statistical analyses

We conducted two analyses to examine the influence of ankle exoskeletons on neuromuscular function. Unless otherwise stated, the values presented in this paper are the means ± s.e. for the participant group. To examine differences in COP parameters, EMG and fascicle length between exoskeleton conditions, we used linear mixed-effects models. For all models, a within-subject design was used, specifying exoskeleton condition (0, 42 and 85 Nm rad^−1^) as the independent variable and including the subject as a random factor using the lme.R function from the nlme package [23] in R (v. 3.6.1, Vienna, Austria).

To examine the influence of exoskeleton assistance on the spatiotemporal relationship between postural control (COP) and muscle activity (EMG), we performed a cross-correlation analysis. To do this, all time-varying data were resampled to a common frequency of 100 Hz. The time-varying COP position signal was cross-correlated with EMG RMS for each of the four muscles, such that at a range of temporal time shifts, a cross-correlation coefficient (*r*) was calculated whereby signals were iteratively shifted with respect to one another in steps of 1 ms up to a maximum phase shift of ±1.5 s. For each of the three conditions and each of the COP–muscle combinations, the cross-correlation time series was combined to provide a mean cross-correlation curve. The maximum cross-correlation value and the respective phase shift of this curve were then found as an indication of the average relationship between the COP and EMG signals. The cross-correlation analysis used in this study was taken across a 20 s time window. We used linear mixed-effects models, as described above, to examine differences in cross-correlation measures between the three conditions. Differences were considered significant at the *p* < 0.05 level.

### 3. Results

#### 3.1. Postural control

**Figure 2.**
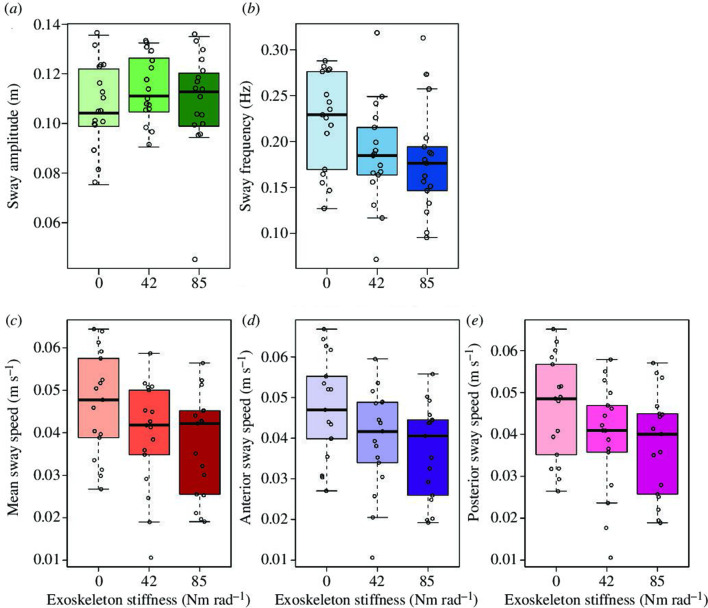
Effect of elastic ankle exoskeleton stiffness on postural sway. Sway amplitude (*a*), sway frequency (*b*), mean COP sway speed (*c*), anterior COP sway speed (*d*), and posterior COP sway speed (*e*) are shown for the three active sway conditions: 0, 42 and 85 Nm rad^−1^. There was a significant decrease in sway frequency as well as mean, anterior and posterior sway speed with increasing exoskeleton stiffness (all *p* < 0.001). Boxplots show 25th and 75th percentiles, the centreline is the 50th percentile and lines represent the max/min values (within the 1.5× interquartile range).

Passive ankle exoskeletons providing plantar flexion assistance induced changes in postural sway during balance tasks. With increasing exoskeleton stiffness, we found a decrease in sway frequency (*p* < 0.001) and a decrease in mean COP speed (*p* < 0.001) (figure 2). Sway frequency decreased from 0.22 ± 0.05 Hz at no assistance (0 Nm rad^−1^) to 0.19 ± 0.06 and 0.18 ± 0.06 Hz at 42 and 85 Nm rad^−1^, respectively. The mean COP speed throughout the sway cycle decreased from 0.047 ± 0.012 m s^−1^ at 0 Nm rad^−1^ to 0.039 ± 0.013 and 0.037 ± 0.012 m s^−1^ at 42 and 85 Nm rad^−1^, respectively.

These changes were related to a decrease in both anterior sway speed (*p* < 0.001) and posterior sway speed (*p* < 0.001) with increasing exoskeleton assistance (figure 2). As expected, sway amplitude did not vary between conditions (*p* = 0.424).

#### 3.2. Fascicle dynamics

**Figure 3.**
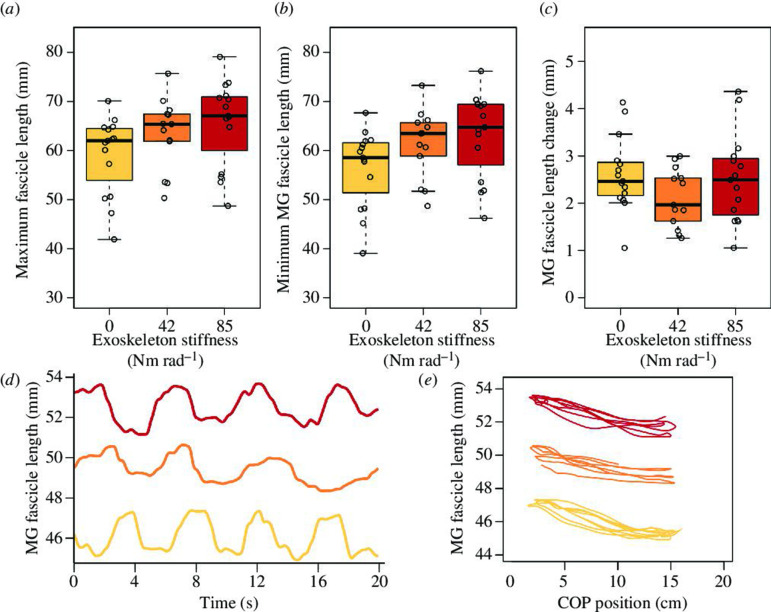
Effect of elastic ankle exoskeleton stiffness on MG muscle fascicle dynamics. Fascicle length increased with increasing exoskeleton stiffness. Box and whisker plots represent maximum fascicle length (*a*), minimum fascicle length (*b*), and total fascicle length change (*c*) during active sway. Time series of MG fascicle length for one representative participant (*d*) and the relationship between COP amplitude and MG fascicle length (*e*) for the three exoskeleton conditions at 0 Nm rad^−1^ (yellow), 42 Nm rad^−1^ (orange) and 85 Nm rad^−1^ (red). There was a significant increase in maximum and minimum fascicle length with increasing exoskeleton stiffness (both *p* < 0.001). Boxplots show 25th and 75th percentiles, the centreline is the 50th percentile and lines represent the max/min values (within the 1.5× interquartile range).

There was a systematic shift in fascicle dynamics to longer operating lengths with increasing exoskeleton stiffness (figure 3). During postural sway, the MG fascicles contracted to shorten during forward sway and lengthened during backwards sway, indicating their paradoxical behaviour during standing balance.

With increasing exoskeleton stiffness, we found that MG fascicles operated on average at 8% (42 Nm rad^−1^) and 11% (85 Nm rad^−1^) longer lengths compared with no assistance (0 Nm rad^−1^) (*p* < 0.001). The shift to longer fascicle lengths is indicated by (i) an increase in maximum fascicle length (*p* < 0.001) from 59.1 ± 8.0 to 63.5 ± 7.3 and 65.7 ± 8.7 mm at 42 and 85 Nm rad^−1^, respectively, and (ii) an increase in minimum fascicle length (*p* < 0.001) from 56.4 ± 7.9 to 61.3 ± 7.0 and 63.1 ± 8.6 mm at 42 and 85 Nm rad^−1^, respectively. However, fascicle length changes remained similar, on average changing length by 2.4 ± 0.78 mm over the whole sway cycle (*p* = 0.117).

#### 3.3. Neuromuscular control

**Figure 4.**
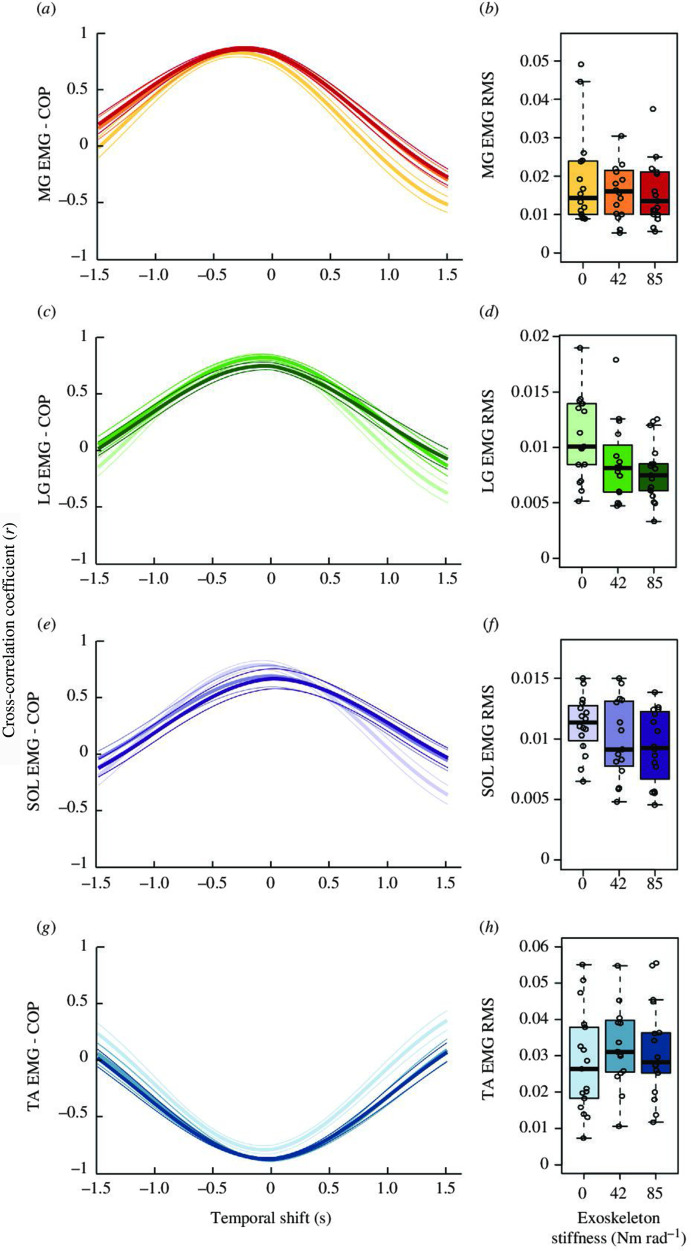
Effect of elastic ankle exoskeleton stiffness on the relationship between muscle activation and postural sway. Grouped mean (thick line) s.e. of the mean (thin line) cross-correlation signals (***r***) calculated between the EMG signals and COP during each condition. The cross-correlation coefficient (***r***) is shown on the *y*-axis, ranging from −1 to +1 in value. The temporal time shift in seconds is located on the *x*-axis and ranges from −1.5 to +1.5 s. Data are shown for each muscle MG (*a*), LG (*c*), SOL (*e*), and TA (*g*) with the average EMG RMS for over the active sway trial for each of the three conditions shown as box and whisker plots (*b*: MG, *d*: LG, *f*: SOL, ***h*: TA**). With increasing exoskeleton stiffness, there was a significant reduction in the correlation coefficient between EMG and COP for the LG (*p* < 0.001) and SOL (*p* = 0.032), but an increase for the TA (*p* = 0.029). There was a significant positive phase shift for the SOL EMG-COP signals (*p* = 0.001) and a decrease in average LG and SOL EMG RMS (both *p* < 0.013) with increasing exoskeleton stiffness. Boxplots show 25th and 75th percentiles, the centreline is the 50th percentile and lines represent the max/min values (within the 1.5× interquartile range).

We also found alterations in the magnitude of ankle plantar flexor muscle activity with increasing exoskeleton assistance. There was a decrease in LG (*p* < 0.001) and SOL (*p* = 0.013) RMS EMG, with no significant changes in MG (*p* = 0.899) or TA (*p* = 0.078) EMG amplitude with exoskeleton assistance (figure 4). Specifically, average LG muscle activation decreased by 20% and 29% at 42 and 85 Nm rad^−1^, respectively, compared with 0 Nm rad^−1^. Similarly, average SOL RMS EMG decreased by 11% and 16% at 42 and 85 Nm rad^−1^, respectively, when compared with the no assistance condition.

To determine whether passive ankle exoskeletons influence the relationship between neuromuscular control and postural sway, we cross-correlated the RMS EMG for each muscle with COP (figure 4). It is apparent that during active sway, there was a strong positive correlation between muscle activation and COP in the ankle plantar flexors MG, LG and SOL (range: 0.74–0.88) and a strong negative correlation in the dorsiflexor TA (range: −0.81 to −0.89). The positive correlation for MG, LG and SOL indicates that these muscles are active during forward sway (in phase), and the negative correlation for TA–COP indicates that the muscle is primarily active during backwards sway (out of phase).

The relationship between muscle activation and COP displacement varied with exoskeleton assistance (figure 4). For the LG (*p* < 0.001) and SOL (*p* = 0.032), we found a reduction in the correlation coefficient with increasing exoskeleton assistance. For the SOL, the peak positive cross-correlation also displayed a slight positive phase shift (*p* = 0.001) from −0.098 ± 0.20 s (0 Nm rad^−1^) to 0.134 ± 0.44 and 0.171 ± 0.41 s at 42 and 85 Nm rad^−1^, respectively. This indicates that the timing of SOL EMG activity was at its greatest just after the most forward point in the sway cycle when performing balance tasks with passive assistance, compared with no assistance. In contrast, for the TA, we observed an increase in the (negative) correlation coefficient with increasing exoskeleton assistance from 0.82 ± 0.14 (0 Nm rad^−1^) to 0.89 ± 0.05 and 0.88 ± 0.06 at 42 and 85 Nm rad^−1^ (*p* = 0.029), suggesting a stronger relationship between TA EMG and postural sway with increasing passive assistance at the ankle. There was no significant influence of exoskeleton assistance on the phase shift between EMG and COP for the MG (*p* = 0.552), LG (*p* = 0.318) or TA (*p* = 0.152).

### 4. Discussion

We asked participants to stand and actively sway in the anterior–posterior direction while wearing an ankle exoskeleton that provided either 0**,** 42 or 85 Nm rad^−1^ of additional rotational stiffness to the ankle joint. The purpose was to observe the influence of exoskeleton plantar flexion torque assistance on sway mechanics and the neuromuscular function of the main ankle muscles during an exaggerated sway task. Our first hypothesis was that the addition of exoskeleton assistance would result in slower swaying, and this was supported by our results (figure 2). We also predicted that plantar flexor EMG would be reduced with increasing exoskeleton stiffness, and this was partially borne out in the EMG results that demonstrated two of three triceps surae muscles had reduced activation with increased exoskeleton stiffness. Furthermore, in partial support of our third hypothesis, MG muscle fascicles operated at increasingly longer lengths with increasing exoskeleton stiffness but did not undergo greater length changes. Finally, our prediction that TA EMG would be greater with increasing exoskeleton stiffness was not backed up by the data, with TA EMG amplitude being unaffected by exoskeleton stiffness.

### 4.1. Control of active sway

Participants were asked to sway so as to match anterior and posterior COP displacement targets, provided via visual feedback. With increasing exoskeleton assistance, the participants were still able to produce the desired COP displacement, but did so at a lower frequency of sway cycles and a slower COP sway speed in both the anterior and posterior directions (figure 2). This suggests that the combined impedance of the exoskeleton and ankle muscles was greater than that of the ankle muscles alone. Therefore, participants did not reduce biological ankle muscle torque contributions sufficiently to match movement speeds in the unassisted condition. Whether this is a result of preference, insufficient adaptation time or an inability to adapt remains unclear, but it is an interesting topic for future research. It is also worth considering that responses may be individualized. Our grouped data capture general trends in sway parameters well, with only two–three participants not reducing sway frequency or increasing speed with increased stiffness (electronic supplementary material, figure S1). This level of consistency seems reasonable for our experiment that was focused on direct measurements of the neuromuscular response to assistance with the goal of identifying changes in function and proposing potential implications for postural control.

Active sagittal sway is a voluntary task that has been shown to be controlled via feedforward strategies involving bursts of pre-emptive neural drive to the agonist plantar flexor muscles [12]. Our results displayed a strong positive cross-correlation between all plantar flexor EMG signals and COP displacement. Without exoskeleton assistance, the phase lag for these cross-correlations was always negative (figure 4), supporting the notion that plantar flexor EMG signal peaks precede forward COP displacement peaks and that anterior sway is under predictive control via neural drive to the plantar flexors [12]. For both the MG and LG, this relationship was maintained when exoskeleton assistance was provided. A slight drop in the strength of cross-correlation was observed for LG, but the coefficient still remained high. However, the cross-correlation for SOL not only became slightly weaker with exoskeleton assistance but also exhibited a shift in the phase lag to become slightly positive as the relative average timing of SOL EMG peak shifted from 10 ms before to 100 ms after the anterior COP displacement peak (figure 4). This suggests that the addition of exoskeleton assistance resulted in the neural drive to SOL being prolonged or delayed such that it reached its maximum activation following the time of peak anterior displacement. This could mean that increasing exoskeleton assistance beyond a certain level causes an important change in how the largest plantar flexor muscle is controlled during forward sway, with muscle activation delayed either deliberately as part of the adaptation to the device or as a result of increased reliance on somatosensory feedback. However, in this study, we were limited to measurements of fascicle behaviour in the MG, and experiments to demonstrate SOL fascicle mechanics that are consistent with this theory are needed. Beck *et al*. [17] have shown that an ankle exoskeleton can significantly alter SOL mechanics during perturbations to standing balance that causes anterior displacement to the body COM. Further work is needed to assess if similar effects exist for planned forward sway actions such as those tested in our study.

### 4.2. Neuromuscular adaptations to exoskeleton assistance

With exoskeleton assistance, fascicle operating length of the MG during the sway task increased (figure 3). This is consistent with the notion that offloading of muscular plantar flexion torque occurs with the exoskeleton [10,11]. Reducing muscle force lowers the tension and stretch applied to the Achilles tendon during anterior sway, and with no change in muscle-tendon unit lengths during the sway cycle, the latter obligates an increase in muscle fascicle length as was observed. Furthermore, with increased exoskeleton spring stiffness, the proportion of total ankle torque contributed by the exoskeleton increases and the contribution from contracting muscle fibres decreases. Therefore, one would expect that progressively increasing exoskeleton stiffness leads to concomitant reductions in muscle force, reduced Achilles tendon stretch and increasing MG fascicle lengths. MG muscle fascicle operating lengths were greater when exoskeleton spring stiffness increased (figure 3), and this is consistent with fascicle length adjustments to exoskeletons during other locomotor activities [10,11]. It was noted that this occurred in the absence of a reduction in MG EMG, specifically. However, the reduced activation in LG and SOL accounts for reduced tension on the Achilles tendon, which is shared between the three muscles and therefore would potentially influence the fascicle length of all three [24]. In general, trends in EMG data were less consistent across participants than for other variables (electronic supplementary material, figure S2), evidenced by lower effect sizes for EMG results. However, the fascicle length data showed highly consistent responses to exoskeleton stiffness between participants (electronic supplementary material, figure S3), and combined with the mechanisms described above, this gives confidence in the general reduction in EMG observed with increasing exoskeleton stiffness.

Increased fascicle operating length when wearing an exoskeleton has the potential to influence the control of standing balance. Although the current task of active sway is predominantly under voluntary control, the current state of a muscle will be important in its reactive response to external perturbations [25]. Increased fascicle length will shift where the muscle operates on the force–length relationship and, in turn, will influence the short-range stiffness of that muscle. Although not entirely responsible for impeding forward motion of the COM [26], short-range stiffness will affect the force produced by ankle plantar flexors in resisting dorsiflexion during postural sway or perturbations to balance. From the present study, it is unknown if the increase in MG fascicle operating length increases or decreases the muscle’s short-range stiffness because we do not know where on the force–length relationship MG is operating. If the fascicles were on the ascending limb, an increase in fascicle length combined with no change in activation (as observed) would be expected to result in greater muscle force and short-range stiffness, owing to a rightward shift in the active force–length relationship. If close to optimum length (*L*_0_) or on the initial part of the descending limb, then the increase in length would reduce active and total muscle force contributions. However, if fascicles were further right on the descending limb, an increase in passive force would be expected to overcome the loss of active force and lead to greater total force with any increase in fascicle length. Given our earlier rationale for increasing exoskeleton stiffness producing increased fascicle length alongside reduced tendon force, a reasonable conclusion is that, without an exoskeleton, the MG muscle fascicles operate close to *L*_0_ and the exoskeleton pushes them to the initial region of the descending limb. However, more careful exploration of this interpretation is required, and the contribution of LG and SOL to Achilles tendon force must be taken into consideration for a complete understanding.

In addition to short-range stiffness, the observed changes in fascicle operating length and/or activation are potentially important for somatosensory feedback from muscle spindles. The simplest interpretation would be that an increased length of extrafusal muscle fibres in MG (assumed from increased fascicle length) would probably have served to increase the operating length of MG muscle spindle intrafusal fibres. Assuming no concurrent adjustments to efferent fusimotor drive from γ-motor neurons, the increased length of intrafusal fibres would be expected to increase afferent activity from muscle spindles [27]. In turn, this would be expected to increase efferent drive. However, we saw no increases in EMG activity with the use of the exoskeleton. This suggests that, even if greater afferent firing was present, the effect on efferent motor unit recruitment was minimal and masked by decreases in descending drive. We also acknowledge the complexities of how spindles operate and how afferent signals can influence motor neuronal drive [27]. Given that reduced muscle force tends to decrease inhibitory 1b afferent firing induced by Golgi tendon organs [28], it could also be that competing changes in spindle and Golgi tendon organ afferent firing cancel each other out. Therefore, the above interpretations are hypotheses in need of experiments designed to test them, but our data suggest a need for work examining how exoskeletons influence spindle function in ankle muscles.

### 4.3. Limitations and future directions

This study represents an exploratory first step into understanding how assistive exoskeletons that provide assistance in parallel to a muscle group influence the neuromuscular control of simple movements such as standing sway. Many of the limitations of this work have raised potential future areas for further research. First, we have recorded fascicle behaviour from one ankle muscle (MG). EMG changes with exoskeleton assistance were not the same in all agonist muscles, and therefore, it may be useful to take measurements of muscle fascicle length from other plantar flexors to capture the full response. Furthermore, the antagonist TA is more likely to be involved in sensory feedback for postural control [29], and therefore, future work should be extended to examine TA muscle responses to delivered assistance. This includes exoskeletons with greater stiffness provision. The present device stiffness was selected to provide torque levels that were well below the biological ankle torques usually generated during standing sway. However, stiffer exoskeleton designs or those intended to support dorsiflexors might influence TA function. Indeed, stiffer exoskeletons worn during more dynamic tasks, where muscles undergo repeated stretch-shortening cycles, could ultimately reduce plantar flexor muscle activation to a level that permits fascicles and spindles to track joint rotation more closely. In this scenario, we might expect to observe greater afferent outputs from plantar flexor spindles. Spindle responses to exoskeleton assistance are also yet to be observed, although there have been attempts to quantify the H-reflex magnitude during assisted gait to examine the excitability of this pathway [30]. We believe it would be valuable to examine acute responses to exoskeleton assistance during sway tasks and further examine exoskeleton influence on responses to perturbations of standing balance. Exoskeletons might induce different feedback in response to joint rotations and muscle stretches because of their effects on short-range muscle stiffness. Some of the changes in muscle activation and fascicle dynamics with increasing exoskeleton stiffness may be a result of the decrease in sway frequency. Future studies could examine this via controlled alterations in sway frequency at a given exoskeleton stiffness.

### 5. Conclusions

In this study, we have described the neuromuscular response of the human ankle plantar flexor muscles to increasing levels of passive assistance from a spring-loaded ankle exoskeleton, during a prescribed standing sway task. Increases in the operating length of MG fascicles, reductions in EMG magnitudes for the SOL and LG, and shifts in the relative timing of SOL activation demonstrate potentially important neuromuscular adaptations to increasing spring stiffness. This opens up a number of highlighted questions for exploration regarding how exoskeletons might influence standing balance and, more broadly, the control of movement.

## Data Availability

The summarized data are accessible at [31]. Electronic supplementary material is available online [32].
